# Dimethyl Fumarate Induces Glutathione Recycling by Upregulation of Glutathione Reductase

**DOI:** 10.1155/2017/6093903

**Published:** 2017-01-01

**Authors:** Christina Hoffmann, Michael Dietrich, Ann-Kathrin Herrmann, Teresa Schacht, Philipp Albrecht, Axel Methner

**Affiliations:** ^1^Focus Program Translational Neuroscience (FTN), Rhine Main Neuroscience Network (rmn^2^) and Department of Neurology, University Medical Center of the Johannes Gutenberg University Mainz, Mainz, Germany; ^2^Department of Neurology, Heinrich Heine Universität Düsseldorf, Düsseldorf, Germany

## Abstract

Neuronal degeneration in multiple sclerosis has been linked to oxidative stress. Dimethyl fumarate (DMF) is an effective oral therapeutic option shown to reduce disease activity and progression in patients with relapsing-remitting multiple sclerosis. DMF activates the transcription factor nuclear factor erythroid 2-related factor 2 (NRF2) leading to increased synthesis of the major cellular antioxidant glutathione (GSH) and prominent neuroprotection* in vitro*. We previously demonstrated that DMF is capable of raising GSH levels even when glutathione synthesis is inhibited, suggesting enhanced GSH recycling. Here, we found that DMF indeed induces glutathione reductase (GSR), a homodimeric flavoprotein that catalyzes GSSG reduction to GSH by using NADPH as a reducing cofactor. Knockdown of GSR using a pool of* E. coli *RNase III-digested siRNAs or pharmacological inhibition of GSR, however, also induced the antioxidant response rendering it impossible to verify the suspected attenuation of DMF-mediated neuroprotection. However, in cystine-free medium, where GSH synthesis is abolished, pharmacological inhibition of GSR drastically reduced the effect of DMF on glutathione recycling. We conclude that DMF increases glutathione recycling through induction of glutathione reductase.

## 1. Introduction

While an increasing number of therapeutic options have been developed to prevent the acute inflammatory insults in multiple sclerosis (MS) there is an urgent need for an effective treatment for the chronic neuronal degeneration occurring afterwards. This is of special importance as this degeneration is thought to be a major factor driving the development of chronic disability in these patients. A promising target for therapeutic interventions is oxidative stress which is prominently involved in neurodegeneration in MS [1–3]. Dimethyl fumarate (DMF) is an effective oral therapeutic, which reduces disease activity and progression in patients with relapsing-remitting MS [4] and psoriasis [5]. DMF and its active metabolite monomethyl fumarate (MMF) [6–8] exert a number of immunomodulatory effects involving increased apoptosis of T cells stimulated with interleukin- (IL-) 2 or anti-CD3 antibodies [9], inhibition of translocation to the nucleus of the nuclear factor kappa B1/p50 (NF-kB1) induced by the cytokines tumor necrosis factor *α* and IL-1*α* [10], and an increased production of protective T helper 2 cytokines IL-4 and IL-5 in CD2/CD8 monoclonal antibody-stimulated peripheral blood mononuclear cells [11]. Besides these immunomodulatory actions, DMF has a prominent antioxidative activity; it first induces short-lived oxidative stress by scavenging the major intracellular antioxidant glutathione (GSH) [12–15]. This results in stabilization and increased levels of the transcription factor nuclear factor erythroid 2-related factor 2 (NRF2) by means of Kelch-like ECH-associated protein 1 (KEAP1) which normally targets Nrf2 for ubiquitination and degradation but loses this ability in response to electrophiles and oxidants [16, 17]. NRF2 then translocates to the nucleus and binds to antioxidant response elements in the promoters of protective genes such as heme-oxygenase-1 [18] and NADPH-quinone-oxidoreductase-1 (NQO1) [19]. This in turn increases the intracellular concentration of GSH [18, 19], rendering the cells more resistant to oxidative stress.

We recently investigated the concentration and time dependence of DMF-mediated protection in neuronal cells and showed that neuroprotective concentrations of DMF depress cytokine production of splenocytes without exerting apoptosis. Neuroprotection was investigated in a model of endogenous oxidative stress, where extracellular glutamate blocks the glutamate-cystine antiporter system Xc^−^ leading to deprivation of cystine and its reduced form cysteine, the rate-limiting substrate for the synthesis of GSH. The subsequent GSH depletion leads to accumulation of reactive oxygen species and cell death by oxidative stress (recently reviewed in [20]). In these neuroprotection assays, the active metabolite MMF was similarly effective but required much longer incubation times to become active [21]. Our results suggest that low doses of DMF and MMF may bring about resistance against oxidative stress and immunomodulation without a need for T cell apoptosis. One important finding of this study was that DMF was still able to raise GSH levels, when the rate-limiting enzyme in glutathione synthesis, glutamate-cysteine ligase, was inhibited or system Xc^−^ activity abrogated by incubation in cysteine-free medium [22]. Therefore DMF can still exert protection, when* de novo *glutathione synthesis is blocked, suggesting enhanced GSH recycling.

The key enzyme that mediates the recycling of GSH is the glutathione reductase (GSR), a homodimeric flavoprotein that catalyzes GSSG reduction to GSH by using NADPH as a reducing cofactor. The GSR promoter contains an antioxidant response element [23], making it a likely candidate for the observed effect. In this contribution, we quantified GSR induction in response to DMF and evaluated the effect of GSR knockdown and pharmacological inhibition on cell death caused by endogenous oxidative stress.

## 2. Materials and Methods

### 2.1. Material

DMF was obtained from Sigma Aldrich and solubilized in dimethyl sulfoxide (DMSO). Cell culture dishes were from Greiner Bio-One. DMEM cell culture medium, sterile phosphate buffers saline, penicillin, streptomycin, L-glutamic acid, L-glutamine 200 mM (100x), sodium pyruvate 10 mM, and Opti-Mem® (1x) were from Gibco Life Technologies. Cell Titer Blue was from Promega. Lipofectamine® RNAiMAX™ reagent was from Invitrogen by Life Technologies and (S)-4-carboxyphenylglycine from TOCRIS.

### 2.2. Cell Culture, Viability Assays, and Glutathione Measurement

We used the hippocampal mouse cell line HT22 which lacks ionotropic glutamate receptors. The cell line had initially been generated as a subclone of the HT4 line [24] selected for a higher susceptibility to glutamate toxicity [25]. HT22 cells were cultured as described [26] and viability quantitated 24 h after glutamate addition by the Cell Titer Blue (CTB) assay (Promega) and normalized to vehicle treatment. Total glutathione was measured enzymatically as described previously [26] and normalized to cellular protein measured by the bicinchoninic acid-based method (Pierce). Glutathione released into the cell culture medium was also quantitated enzymatically after 4 h in cystine-free medium and normalized to total cellular protein; 1,3-bis[2-chloroethyl]-2-nitrosourea (BCNU; Carmustine) was solubilized in ethanol, which was also used as the vehicle control. (S)-4-Carboxyphenylglycine was solubilized in NaOH, which was also used as the vehicle control. Cystine-free medium was prepared by using DMEM, high glucose, w/o glutamine, methionine, cystine supplemented with 1% sodium pyruvate (100 mM), 2% L-glutamine (200 mM), and 3% L-methionine.

### 2.3. siRNA Transfection

Mission® esiRNA against mouse KIF11, FLUC, and mouse GSR, L-methionine, and BCNU were obtained from Sigma Aldrich. Transfections were performed with Lipofectamine according to the manufacturer's protocol (Life technologies). Briefly, cells were transfected with 1600 ng esiRNA in 24-well plates and replated 48 h later in a density of 2500 cells per well into 96-well plates.

### 2.4. Immunoblotting

Immunoblotting was performed as previously described [26] using antibodies against GSR (N-Term) antibody (1 : 1000; Antikoerper-online.de, ABIN406391) and anti-actin antibody (1 : 4000; Millipore MAB1501). Secondary antibodies were anti-rabbit IgG (H + L) (DyLight™ 800 Conjugate) and anti-mouse IgG (H + L) (DyLight™ 680 Conjugate) from Cell Signaling Technology™ (1 : 30000).

### 2.5. Quantitative Real-Time PCR

RNA extraction, reverse transcription, and quantitative real-time PCR were performed as previously described [26] using Fam/Dark-quencher probes from the Universal Probe Library™ (Roche) or individually designed Fam/Tamra probes (MWG). Beta-actin and HPRT served as endogenous control genes and showed no differential expression after incubation with DMF. Primer and probe sequences can be obtained from the authors.

### 2.6. Statistical Analysis

Statistical analysis was performed using spreadsheet (Microsoft Excel) and Prism (Graphpad) software. Multiple group analyses were conducted with two-way ANOVA and Bonferroni or Dunnett's* post hoc* test, comparison of two groups with two-tailed *t*-test. *p* values <0.05 were considered significant.

## 3. Results and Discussion

### 3.1. Cytoprotective Concentrations of DMF Induce the Expression of Glutathione Reductase

**Figure 1:**
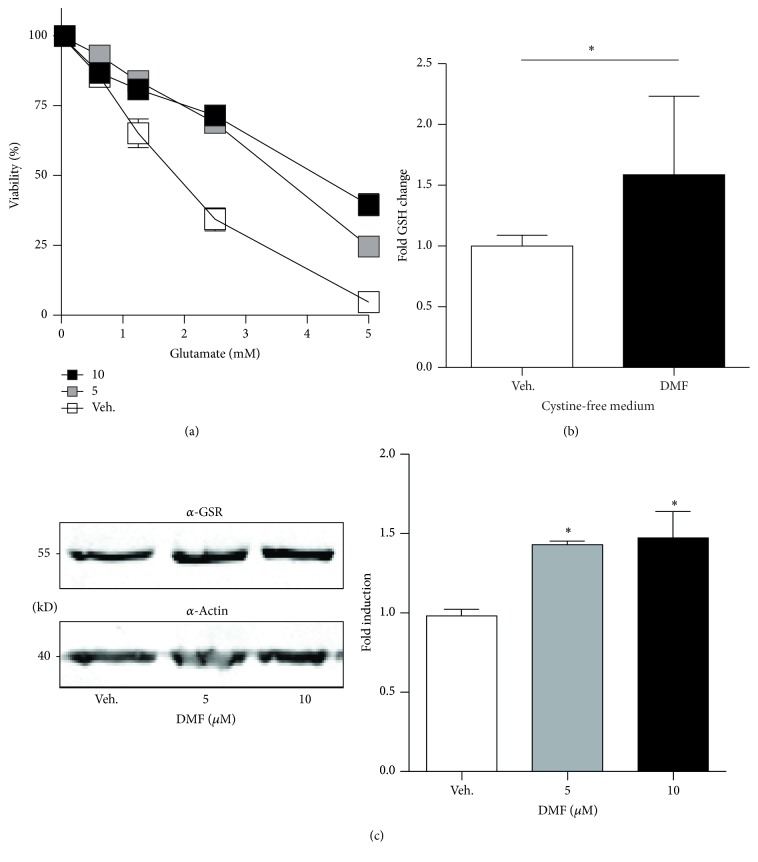
Cytoprotective concentrations of DMF induce the expression of glutathione reductase. (a) HT22 cells were treated for 24 h with the indicated concentrations of DMF before addition of glutamate. Viability was quantified 24 h later by the CTB assay. (b) DMF still elevates cellular GSH when GSH synthesis is blocked by incubation in cystine-free medium for another 24 h before intracellular GSH was measured enzymatically. (c) Cells were treated with DMF for 24 h and the abundance of GSR was quantitated by immunoblotting. Actin served as loading control. Molecular mass is indicated. The bar graphs represent the means ± SD of three independent experiments, ^*∗*^ *p* < 0.05, two-way ANOVA, and Tukey's* post hoc* test.

We first reproduced our findings that DMF protects against glutamate toxicity and found that 5 and 10 *μ*M DMF induced a robust protection within 24 h (Figure 1(a)) as previously described. This protection involved an increase in GSH content even in conditions where no GSH can be synthesized because of a lack of the essential building block cystine (Figure 1(b)). This indicates that the increase in GSH observed here is due to an increase in glutathione recycling. In line with this, we indeed observed an increase in the abundance of GSR in cells treated with the protective concentrations of DMF, 5 and 10 *μ*M, as shown by immunoblotting with an antibody specific for GSR and compared to *β*-actin as loading control (Figure 1(c)). DMF therefore induces the expression of the key enzyme involved in GSH recycling, GSR.

### 3.2. Identification of Small Interfering RNAs against GSR

**Figure 2:**
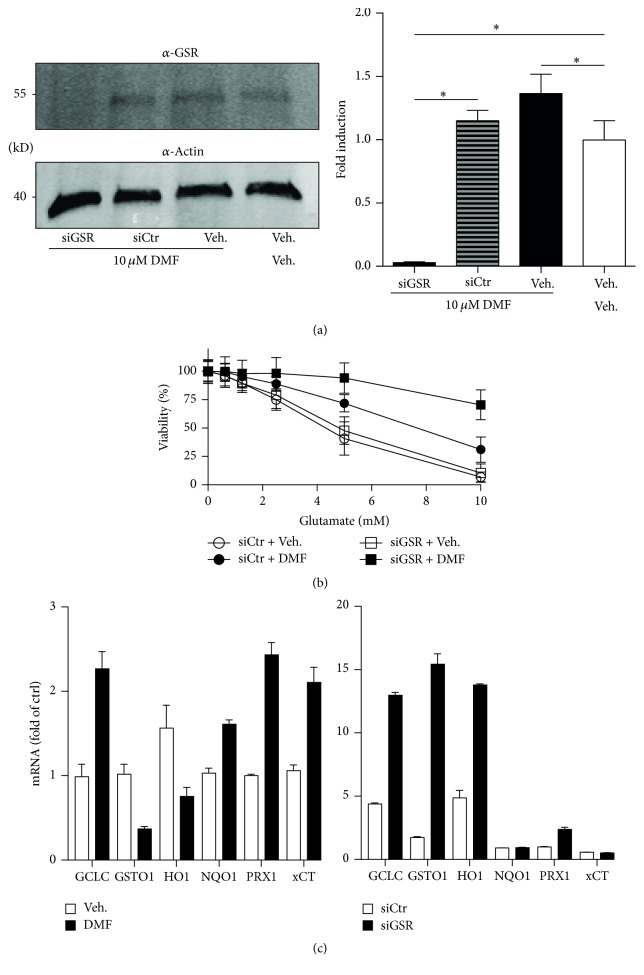
Knockdown of GSR boosts the protective effect of DMF by inducing a synergistic set of antioxidant response genes. (a) Cells were transfected with siRNAs against GSR (siGSR) or luciferase (siCtr) 24 h before addition of 10 *μ*M DMF for an additional 24 h. The same amount of protein lysates was blotted and stained with antibodies against GSR or actin as loading control. The molecular weight is indicated. The bar graphs represent the means ± SEM normalized to vehicle of 3 different blots. (b) DMF was added 24 h after transfection of the indicated siRNAs and 10 mM glutamate 24 h after DMF. Viability was quantified by CTB assays again 24 h later. The bar graphs represent the mean ± SEM of three experiments done in triplicate. (c) DMF treatment or siGSR transfection induces mRNA expression of a synergistic set of transcripts involved in the antioxidant response. Cells were treated for 24 h with 10 *μ*M DMF or vehicle or prepared 48 h after transfection with siGSR and siCtrl and mRNA quantitated by real-time PCR using *β-actin* and* hprt* as endogenous controls. ^*∗*^ *p* < 0.05, two-way ANOVA, and Tukey's* post hoc* test.

To clarify the contribution of GSR to the protection conferred by DMF we decided to knockdown GSR with endoribonuclease-prepared small interfering inhibitory RNAs (esiRNAs) and pools of siRNAs resulting from cleavage of long double-stranded RNA with* Escherichia coli* RNase III. We transfected HT22 cells with esiRNA against GSR or against luciferase as control. After 24 h, 10 *μ*M DMF or vehicle was added and after again 24 h protein lysates were used for immunoblotting with antibodies against GSR or actin as loading control. Untransfected cells treated with DMF or vehicle served as additional controls. The esiRNA against GSR indeed completely abolished GSR expression (Figure 2(a)). DMF was not able to induce GSR expression in the presence of GSR-specific esiRNAs whereas GSR was still expressed in the presence of esiRNA directed against luciferase. We concluded that esiRNA-induced knockdown could serve as a tool to elucidate the contribution of GSR to DMF-mediated protection against oxidative stress.

### 3.3. Knockdown of GSR Boosts the Protective Effect of DMF by Inducing a Synergistic Set of Antioxidant Response Genes

We transfected the cells with esiRNA against GSR and control esiRNA in 6-well-plates. 24 h later the cells were treated with DMF or vehicle and again 24 h replated into 96-well-plates where they were then exposed to 10 mM glutamate for an additional 24 h. We observed two things; first, esiRNA against GSR induced a protection by itself and second, this even boosted the protection conferred by 10 *μ*M DMF (Figure 2(b)). We hypothesized that the lack of GSR over 48 h before the additional treatment with glutamate probably also induces the antioxidant response synergistically to DMF which increases nuclear Nrf2 protein levels [21].

To clarify the observed synergistic effect of the combination of DMF and GSR knockdown, we quantitated the expression of genes belonging to the antioxidant response battery in DMF- and siGSR-treated cells and their respective controls. This indeed proved that both treatments result in the induction of a synergistic set of antioxidant transcripts. Only the catalytic subunit of the glutamate-cysteine ligase (GCLC) and peroxiredoxin 1 (PRDX1) was upregulated in both sets, whereas glutathione* S*-transferase omega 1 (GSTO1) and heme-oxygenase 1 (HO-1) were downregulated in DMF-treated but upregulated in siGSR-treated cells. NADPH-quinone-oxidoreductase-1 (NQO1) and xCT (also known as SLC7A11) showed the opposite pattern (Figure 2(c)).

### 3.4. Pharmacological Inhibition of Glutathione Reductase Is Also Protective When Preincubated for 24 h

**Figure 3:**
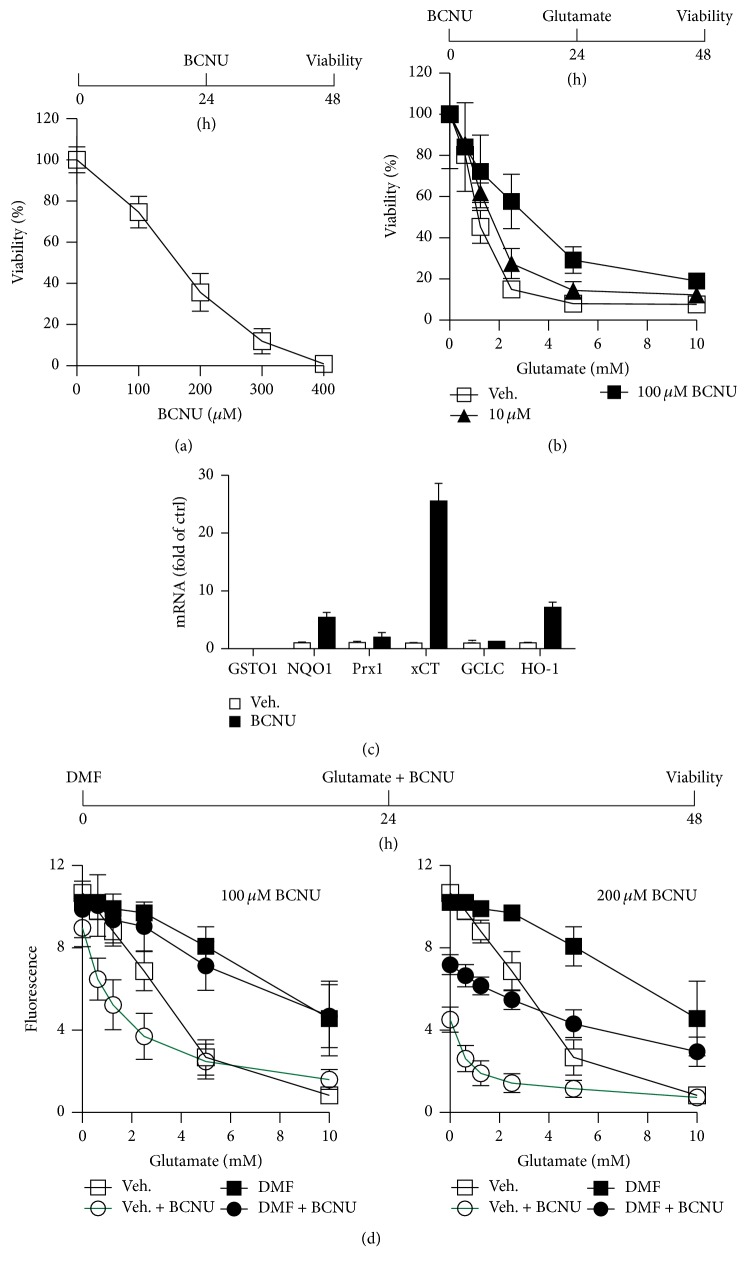
Pharmacological inhibition of glutathione reductase with BCNU is also protective when preincubated for 24 h. HT22 cells were (a) treated for 24 h with the indicated concentrations of BCNU or (b) pretreated with the indicated concentrations of BCNU for 24 h before addition of glutamate at the indicated concentrations for another 24 h. Afterwards, viability was quantified by the CTB assay (a and b). (c) Cells were treated for 24 h with 50 *μ*M BCNU or vehicle and mRNA quantitated by real-time PCR using*β-actin* and* hprt* as endogenous controls. (d) HT22 cells were treated with 10 *μ*M DMF for 24 h before simultaneous addition of BCNU and glutamate in the indicated concentrations. Viability was quantified 24 h later by the CTB assay.

BCNU (Carmustine) is an antitumor, DNA-alkylating agent, which inhibits cellular glutathione reductase activity [27]. A pharmacological agent should theoretically inhibit GSR without delay and allow a more precise analysis of the contribution of glutathione recycling in the protective effect of DMF with the important caveat of less specificity because most inhibitors inhibit more than one enzyme. BCNU concentration-dependently provoked cell death in HT22 cells with an LD50 of approximately 200 *μ*M (Figure 3(a)). As expected, much lower concentrations of 10 and 100 *μ*M again elicited a protection (Figure 3(b)) again mediated by the induction of some antioxidant transcripts, most prominently the cystine-glutamate antiporter xCT (SLC7A11), HO-1, and NQO1 (Figure 3(c)).

We then tried to attenuate the protective effect of DMF preincubation by a simultaneous exposure of the cells to glutamate, which in these cells inhibits cystine import and therefore leads to glutathione depletion, and BCNU. We observed only a very minor, not statistically significant reduction in viability in cells treated with 100 *μ*M BCNU. Cells not pretreated with DMF but exposed to glutamate and BCNU, in contrast, were significantly more prone to cell death (Figure 3(d)). This means that DMF even protects against a combined assault with an agent that inhibits* de novo* glutathione synthesis, glutamate, and glutathione recycling, BCNU, suggesting additional, not yet known protective mechanisms induced by DMF. At a higher concentration of BCNU, 200 *μ*M, an antiproliferative effect prevailed (Figure 3(d)).

### 3.5. Pharmacological Inhibition of Glutathione Reductase Inhibits the Positive Effect of DMF on Glutathione Recycling in Cystine-Free Medium

**Figure 4:**
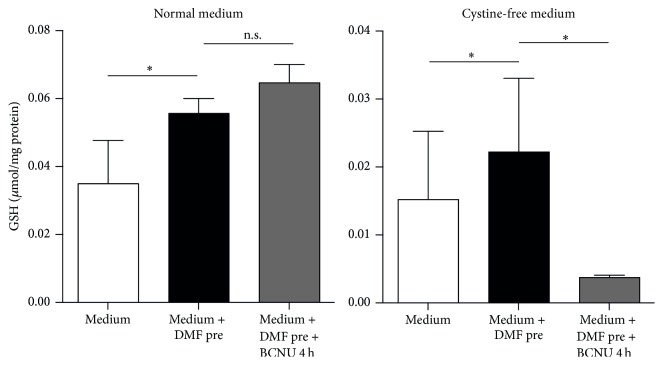
Pharmacological inhibition of glutathione reductase inhibits glutathione recycling in cystine-free medium. DMF still elevates cellular GSH when GSH synthesis is blocked by incubation in cystine-free medium. HT22 cells were treated for 24 h with 10 *μ*M DMF (black bars) or vehicle (white bars) and then exposed to cystine-free medium for another 4 h in the presence of 50 *μ*M BCNU (grey bars) before intracellular GSH was measured enzymatically. Graphs of all experiments represent the means ± SD of three independent experiments performed in triplicate. ^*∗*^ *p* < 0.05, two-way ANOVA, and Tukey's* post hoc* test.

We concluded from these experiments that we could only study the inhibitory effect of BCNU on DMF-mediated glutathione recycling under conditions where (1)* de novo* glutathione synthesis is inhibited and (2) the time of incubation with BCNU is not long enough to allow the induction of gene transcription. We therefore pretreated cells with 10 *μ*M DMF which increased GSH concentration in both normal medium and cystine-free medium as shown in Figure 1(b) and as previously reported [21]. The presence of BCNU for 4 h in the cystine-free medium, however, completely abolished the GSH recycling mediated by DMF (Figure 4). These experiments prove that part of the positive effect of DMF on glutathione content is indeed mediated via increased glutathione recycling.

## 4. Conclusions

Our major finding is that DMF indeed increases glutathione recycling by induction of GSR. Our studies were hampered by the fact that both knockdown and inhibition of GSR induced a strong antioxidant response by itself. To study the effect of GSR inhibition on glutathione recycling alone, incubation in cystine-free medium can be used to block the* de novo* synthesis of GSH and avoid confounding effects of GSR inhibition.

## Abbreviations

BSO: Buthionine sulfoximine
CTB: Cell Titer Blue
DMF: Dimethyl fumarate
DMSO: Dimethyl sulfoxide
GCLC: Glutamate-cysteine ligase, catalytic subunit
GSH: Glutathione
MS: Multiple sclerosis
NF-*κ*B: Nuclear factor kappa B
Nrf2: Erythroid 2-related factor 2
NQO1: NADPH-quinone-oxidoreductase-1
S4-CPG: (S)-4-Carboxyphenylglycine.
